# Management of Elderly Colorectal Cancer Patients: A Comprehensive Review Encompassing Geriatric Assessment

**DOI:** 10.3390/cancers17203336

**Published:** 2025-10-16

**Authors:** Alessandra Boccaccino, Martina Cassaniti, Daniele Rossini, Laura Faccani, Chiara Casadio, Stefano Tamberi

**Affiliations:** 1 Oncology Unit, Santa Maria delle Croci Hospital—AUSL Romagna, 48121 Ravenna, Italy; alessandra.boccaccino@auslromagna.it (A.B.); laura.faccani2@auslromagna.it (L.F.);; 2 Oncology Unit, University Hospital of Ferrara, 44124 Ferrara, Italy; 3 Department of Experimental and Clinical Medicine, University of Florence, 50121 Florence, Italy; 4 Oncology Unit, IRCSS Azienda Ospedaliero-Universitaria di Bologna, 40138 Bologna, Italy; 5 Department of Medical Sciences (DIMEC), University of Bologna, 40126 Bologna, Italy

**Keywords:** colorectal cancer, geriatric oncology, elderly patients

## Abstract

Colorectal cancer (CRC) is a common tumor, and its incidence increases with age. Elderly CRC patients constitute a daily challenge for oncologists when deciding if they are worthy of active treatments and of which kind, as data from the literature are incomplete or even lacking in specific subsets. To fill this gap, we conducted a narrative review that included not only studies dedicated to the elderly but also extracted elderly groups from major clinical trials. Results in terms of harm and benefit were dissected according to frailty categories (fit, vulnerable, unfit). As it emerged, the management of elderly CRC patients should be based not on age alone, but rather on their frailty level, as assessed by Comprehensive Geriatric Assessment (CGA), which should be implemented in trials and clinical practice. Elderly patients should be treated similarly to young patients in the first and subsequent lines, without precluding the use of innovative drugs. Vulnerable elderly individuals should receive personalized schedules that focus more on symptoms and quality of life; for frail patients, supportive care alone is often a valid option.

## Simple Summary

Colorectal cancer (CRC) mainly affects patients aged over 65 years. Given the heterogeneity in the aging process, it is fundamental to accurately stratify older CRC patients through geriatric assessment (GA) in order to administer the most effective treatment, not based on age alone but rather according to the individual’s frailty level. However, the elderly are underrepresented in CRC trials; in addition, GA is often missing even in studies dedicated to the elderly. Consequently, oncologists’ daily decisions are often based on a few literature data with a low level of evidence. Therefore, we decided to perform a comprehensive literature review to evaluate the benefits and harms of administering the current standard of care to elderly CRC patients, ranging from localized to metastatic disease. Our findings led to practical suggestions on how to manage elderly CRC patients based on GA alongside canonical tumor characteristics.

## 1. Introduction

Colorectal cancer (CRC) is among the most prevalent malignancies worldwide [1]. Multiple risk factors contribute to the development of CRC, which can be broadly categorized as either modifiable or non-modifiable. Age stands out as the most significant non-modifiable risk factor, with incidence rising markedly over time [2]. Approximately two-thirds of CRC cases are diagnosed in individuals over the age of 65, positioning the elderly as a particularly vulnerable demographic, underscoring the need for treatment strategies adapted for them [3].

Conversely, elderly patients are frequently underrepresented in clinical trials. Defining “elderly” in clinical practice is inherently challenging due to the considerable individual variability in the aging process, often rendering chronological age a mere number that fails to accurately match biological age. Traditionally, the threshold for “old age” has been set at 65 years. However, the age of 70 is now more commonly used as the cut-off to indicate the onset of clinical senescence, since age-related decline typically accelerates beyond this point [4,5]. Moreover, from the age of 85 onwards, individuals experience an even faster functional decline, increasing their vulnerability [6].

To overcome the discrepancy between chronological and biological age, a comprehensive and individualized assessment encompassing physiological reserve and functional status is advisable when approaching elderly patients with cancer. Factors such as frailty, multimorbidity, cognitive and functional impairments, as well as psychosocial and socioeconomic background, must all be integrated into the therapeutic plan. In this context, the Comprehensive Geriatric Assessment (CGA) emerges as a valuable tool.

In this narrative review, we examined the literature on the management of elderly CRC patients. JBI’s critical appraisal checklist for textual evidence has been applied to assess the quality of the included papers (Supplementary Materials) [7]. More in detail, we tried to evaluate how many elderly were included in the trials on which the current CRC treatment algorithm is based; how many elderly dedicated protocols were conducted for each disease setting; and how elderly patients were screened and selected, particularly focusing on the implementation of CGA. Also, we tried to estimate the expected treatment benefit in terms of oncological outcomes and toxicities among elderly patients, both overall and according to their level of frailty.

Our aim is to raise awareness of the clinical unmet needs of elderly mCRC patients and ultimately contribute to the improvement of their management.

## 2. Geriatric Assessment in Clinical Practice

CGA is a multidimensional and multidisciplinary approach that deeply dissects functional and nutritional status, cognitive and psychological sphere, comorbidities, and social support networks [8]. This assessment is crucial for achieving a holistic understanding of elderly cancer patients, allowing clinicians to identify specific vulnerabilities and thereby prevent further deterioration in functional status as a consequence of oncological treatments. Major oncology Societies recommend incorporating CGA into clinical practice [9] as numerous studies demonstrated its capacity to inform and modify treatment decisions [10].

Based on the CGA, elderly cancer patients are stratified into three categories: fit, vulnerable, and frail. Each group is characterized by distinct life expectancy, oncological outcomes, and risk profiles related to treatment [11,12,13]. An unfavorable CGA evaluation is also correlated to poorer results on Quality of Life (QoL) questionnaires administered before, during, and after treatment [14].

Fit patients are fully independent in their daily activities (ADLs) and have no relevant comorbidities; they are considered eligible for standard therapies like younger individuals. Frail patients are typically aged 85 years or older, dependent in ADLs, and present with three or more comorbidities and at least one geriatric syndrome; for these patients, supportive care is often the preferred option. Vulnerable or “in between” patients need assistance in at least one ADL and have one or two comorbidities; they may benefit from adjusted-dose treatment or monotherapy [15].

While the CGA is the most comprehensive tool for assessing elderly cancer patients, its complexity and the time required for its administration often limit its use in routine clinical practice. For this reason, several rapid screening tools have been developed to efficiently identify patients who are most likely to benefit from a comprehensive evaluation. The Geriatric-8 (G8) and the Vulnerable Elders Survey-13 (VES-13), with their high sensitivity in detecting geriatric impairment, are among the most used. The G8 consists of eight items assessing CGA key domains: A total score of ≤14 indicates potential frailty and the need for a CGA. The VES-13, composed of 13 questions related to age, self-rated health, and physical function, assigns a cumulative score where values ≥ 3 suggest a high level of vulnerability [16,17]. The age-adjusted Charlson comorbidity index (age-CCI) is another frequently used scale that assesses comorbidity to estimate 5-year survival. A higher score strongly correlates with increased mortality, overall and among cancer patients [18].

## 3. Adjuvant Chemotherapy

The current standard of care for patients with resected stage III and high-risk stage II CRC is a fluoropyrimidine plus oxaliplatin adjuvant chemotherapy for three or six months according to risk stratification, while the role of adjuvant therapy in intermediate stage II is still debated [19].

Adjuvant FOLFOX/XELOX for stage III patients is based on the pivotal trials MOSAIC, NO16968/XELOXA, and NSABP C-07 [20,21,22]. The MOSAIC trial (N = 2246) enrolled stage II and III CRC patients up to 75 years of age. Thirty-five percent of patients were ≥65 years old, while only 15% were 70–75 years old. Treatment tolerance was similar between older (≥70 years) and younger patients, while the addition of oxaliplatin to fluorouracil-leucovorin (5-FU/LV) did not significantly improve disease-free survival (DFS) (*p* = 0.710), time to relapse (TTR) (*p* = 0.14), and overall survival (OS) (*p* = 0.66) among the elderly. However, no significant interaction was found between treatment results and age [23,24].

Similarly, out of 2409 stage II and III patients eligible for the NSABP C-07 trial, only 30% were ≥65 years old, and 14% ≥70. Overall survival was not improved among the elderly (≥70 years) receiving oxaliplatin (HR for OS 1.18, *p* = 0.30), or rather it numerically decreased by 4.7% at 5 years, possibly due to the not-negligible toxicity of the FLOX regimen [22,23,24,25].

The XELOXA/NO16968 trial enrolled 1886 stage III CRC patients without an upper age limit (range 22–83 years), including 409 (22%) patients ≥ 70 years. The addition of oxaliplatin provided a significant DFS advantage and tended towards a better OS across all subgroups, including patients ≥ 65 years old, albeit to a lesser extent among the eldest (HR for 3-year DFS < 70 years: 0.79; ≥70 years: 0.87). No interaction of age by treatment was found (*p* = 0.622). An overall higher rate of G3–4 (65% vs. 57%) and serious adverse events (30% vs. 17%), and toxicity-related withdrawals (30% vs. 16%) were reported among patients ≥65 years receiving oxaliplatin [21,26].

A pooled analysis of ACCENT and IDEA trials comprising 17,909 patients with 24.2% ≥70 years and 8.1% ≥75, showed that high-risk features (e.g., T4, right primary tumor, *BRAF* V600E mutations) are more commonly found among older (≥70 years) patients with resected stage III CRC undergoing adjuvant treatment compared to the younger counterpart. Also, treatment interruption, dose reductions, or oxaliplatin discontinuation were more frequent, particularly for the 6-month regimen. In the multivariable model, all the oncological outcomes were worse for the elderly except for TTR (*p* = 0.18). Interestingly, no difference was found in terms of DFS (*p* = 0.51) and OS (*p* = 0.65) between elderly patients receiving a 3- or 6-month regimen, even when stratified per risk group [27].

Other pooled analyses and reviews support the benefit of adjuvant chemotherapy for resected stage III CRC patients with or without oxaliplatin, despite age, but it should be acknowledged that the elderly included in those studies represented a minor portion of patients, were mostly younger than 80 years, and fit for a clinical trial [27,28], differently from daily practice. However, the proportion of the elderly enrolled in clinical trials seems to have increased over time [27] and observational studies too suggest that adjuvant chemotherapy can improve survival in elderly (≥70 years) patients with resected stage III CRC [29]. On the contrary, the increased risk of high-grade toxicities with oxaliplatin raises concerns about its administration to the elderly, alongside the limited or absent survival benefit suggested from other studies [28,30].

Randomized interventional trials specifically designed for the elderly are needed to better clarify the actual evidence. The phase III PRODIGE 34—ADAGE trial prospectively evaluated the administration of adjuvant chemotherapy to the elderly. Resected stage III CRC patients aged ≥ 70 years were divided in two cohorts according to CGA at baseline: fit patients (group 1), who are considered eligible for doublet chemotherapy, were randomized to fluoropyrimidine alone (arm A) or plus oxaliplatin (arm B) for 6 months; frail patients (group 2), who are unsuitable for oxaliplatin, were randomized to six months fluoropyrimidine monotherapy (arm D) or observation only (arm C). Out of 491 patients enrolled at the time of the preliminary tolerance analysis, 378 were assigned to group 1. Compared to group 1, frail patients (group 2) were older (median age: 83 vs. 76 years), with a worse performance status (ECOG PS 2–3: 24% vs. 4%), and were more often diagnosed after obstruction or perforation. Also, high microsatellite instability/deficient mismatch repair (MSI-H/dMMR) was more common (25% vs. 17%).

As expected, more high-grade AEs were registered in arm B (N = 189) than arm A (N = 189) (58% vs. 26%) thus without sensibly affecting treatment delays and duration. Frail patients receiving fluoropyrimidine (arm C, N = 56) experienced more high-grade AEs than fit patients treated with the same regimen (G3–5 AEs: 40% in arm C vs. 26% in arm A), and more often delayed (60% vs. 56%) or precociously interrupted (38% vs. 17%) chemotherapy, or did not start treatment at all (16% vs. 2%). Interestingly, treatment discontinuation due to toxicity was also more frequent for arm D than arm B, even with the higher rate of AEs in arm B, highlighting the difference in patients’ resiliency according to their frailness, alongside physicians’ confidence in continuing treatment in unfit patients. Efficacy results from PRODIGE 34—ADAGE are awaited [31].

For elderly patients with resected stage II CRC, evidence supporting adjuvant chemotherapy is even less. In a meta-analysis including 426 elderly (≥70 years) stage II CRC patients, no significant gain in terms of OS (RR 0.73, 95% CI 0.51–1.06, *p* = 0.09) was reported for those who received adjuvant chemotherapy compared to those who did not, although the quality of evidence was low [29]. In a post hoc analysis of the MOSAIC trial, the addition of oxaliplatin to 5-FU/LV showed no significant benefit in terms of DFS or OS in patients aged 70–75 years, including 15% low-risk and 25% high-risk stage II [24].

Data on adjuvant chemotherapy in the elderly remain mixed and partially inconclusive. Although evidence supporting adjuvant treatment in stage II disease remains unfavorable, the scenario in stage III is more nuanced. These results suggest that the effect of adjuvant treatment does not differ according to age per se and that the absence of a clear benefit among the elderly may be due to early adjuvant discontinuation, less intensive treatments at the time of disease relapse, and competing causes of death. A CGA stratification could help select older patients who may really benefit from adjuvant treatment, with limited adverse events even with oxaliplatin doublet (Table 1).

## 4. Metastatic Disease

The current standard first-line treatment for microsatellite stable/proficient mismatch repair (MSS/pMMR) mCRC patients is essentially represented by a fluoropyrimidine (5-FU or capecitabine) combined with irinotecan and/or oxaliplatin, together with either the anti-VEGF bevacizumab or an anti-EGFR, according to patient and tumor characteristics [32]. However, the optimal treatment approach for mCRC patients aged 70 years and above remains uncertain, due to the limited inclusion of elderly individuals in clinical trials and the heterogeneous health conditions typically observed in this population. Furthermore, prolongation of survival may not be the primary expectation for older patients and their caregivers; rather, the main goals often include symptom control, prevention of complications, and maintenance of functional independence, ultimately aiming at preserving a good quality of life (QoL) [33].

First-line recommendations are summarized in Table 2.

### 4.1. First-Line Treatment

#### 4.1.1. Chemotherapy

Combining 5-FU with irinotecan (FOLFIRI) or oxaliplatin (FOLFOX) results in superior clinical responses and improved survival outcomes compared to 5-FU monotherapy among mCRC patients overall [33]. A meta-analysis of 5 phase III trials comparing doublets (FOLFIRI/FOLFOX) to 5-FU alone among 1225 elderly (≥70 years) mCRC patients, showed a modest superiority of both doublet regimens in terms of progression-free survival (PFS) (HR = 0.82, *p* = 0.003), without a corresponding benefit in OS (HR = 1.00, *p* = 0.962) [34].

Regarding the addition of oxaliplatin, several studies support its use. In this context, prospective phase II trials enrolling patients ≥ 70 years old [35,36,37,38,39] and post hoc subgroup analysis from large, randomized phase III trials [40,41] confirmed the efficacy and activity of XELOX/FOLFOX regardless of age, with improvements in QoL [39] and geriatric scales [38] over time. Oncological outcomes were not influenced by age, geriatric scores [38] or number of comorbidities [36]. However, it should be noted that elderly patients were eligible for those studies only if in good general condition with an ECOG PS ≤ 2 [35,36,37,38].

Additionally, the vast majority of the patients from prospective trials resulted fit at baseline CGA, being essentially independent in ADL/IADL and with few relevant comorbidities [35,37,38]. From a safety perspective, elderly patients treated with oxaliplatin more frequently experienced high-grade toxicities than younger patients, particularly neutropenia and thrombocytopenia, while the incidence of other common adverse events, such as diarrhea, was similar between groups, and peripheral neuropathy was generally of low grade [33,41]. The significance of the relationship between age and higher risk of AEs increased when age was analyzed as a continuous variable in the overall population [33]. However, among elderly patients, age, geriatric scores, and number of comorbidities did not appear to be correlated with the occurrence of toxicities [35,36].

For patients unfit for standard regimens but still suitable for combination treatment, namely vulnerable elderly, tailored schedules of oxaliplatin-based doublets have been proposed. For example, a first-line treatment with 5-FU and oxaliplatin at reduced dose or fractionated over two days may spare high-grade neuropathy and hematologic toxicity, while achieving tumor response and prolonging survival to 1 year or more [38,42].

Finally, oxaliplatin should not be administered to frail patients, as noted in the phase II MRC FOCUS2 study, which randomized 459 elderly and frail patients to receive a fluoropyrimidine with or without oxaliplatin. The median age of patients was 74 years (range 35–87), with 43% >75 years and 13% >80 years. The first cycle was administered at 80% of the standard dose, with the possibility of subsequent escalation according to tolerance. Only 14% of patients were able to tolerate the full dose, while almost half needed a further reduction or interruption. The addition of oxaliplatin did not result in a significant prolongation of PFS (*p* = 0.07) or OS (*p* = 0.91) but was associated with QoL deterioration (*p* = 0.04). More interestingly, the MRC FOCUS2 trial assessed overall treatment utility (OTU), a composite measure that summarizes the harm and benefit from treatment. The probability of worse OTU was dependent not only on age per se but also on symptoms, ECOG PS, and extension of disease [43], reaffirming the need for baseline CGA to refine patients’ selection before administering or precluding combination first-line treatment.

By contrast, the addition of irinotecan to fluoropyrimidine-based regimens has yielded more modest results in elderly patients. In a meta-analysis including 2691 mCRC patients with 22% ≥70 years (15% 70–74 years, 6% 75–79 years, and less than 1% 80 years or more), FOLFIRI compared to 5-FU monotherapy improved PFS both in young and old patients, while only young patients derived significant benefit in terms of OS. However, no interaction for response rate (*p* = 0.33), PFS (*p* = 0.84), and OS (*p* = 0.61) between treatment arm and age was found, even when age was analyzed as a continuous variable [42]. Conflicting results on the additional benefit of irinotecan came from prospective phase II and III trials dedicated to elderly patients [44]. The phase III FFCD 2001–02 trial randomized elderly patients (≥75 years) to 5-FU plus or less irinotecan and stratified them by CCI and Karnofsky performance status (KPS). Around half of the patients were at least 80 years old, while only a third had a low KPS and less than 10% a scarce CCI. Age, KPS, and CCI had no predictive value on treatment efficacy [45]. A higher incidence of grade 3–4 toxicities—particularly diarrhea, nausea, vomiting, and neutropenia—has frequently been associated with the addition of irinotecan to 5-FU in elderly patients [34,42,46,47]. However, some studies have reported manageable toxicity profiles and even slight improvements in QoL [48] suggesting that irinotecan-based therapy may be feasible in selected patients aged ≥ 70 years. Nevertheless, impaired geriatric assessment scores [49] and a more advanced age [45] appear to be correlated with a higher risk of severe adverse events.

Despite sometimes conflicting results and evidence that often comes from post hoc subgroup analysis or small prospective studies, taken together, these results suggest that elderly fit patients should not be excluded from combinational treatment when anticipated clinical benefit is correctly weighed against potential severe toxicities.

Actually, elderly patients who are in very good general conditions and without relevant comorbidities deserve an intensification of chemotherapy, even up to a triplet combination. A pooled analysis including 784 fit elderly (defined as 71–75 years old with ECOG PS = 0) mCRC patients receiving first-line FOLFOXIRI with or without targeted agents in the context of clinical trials, showed activity and efficacy in line with the overall population (ORR 65%; mPFS 10.6 months; mOS 23.7 months), without additional safety concerns [50].

On the contrary, the best approach for previously [31,50] untreated, unfit elderly mCRC patients remains not clearly defined, mainly due to the limited representation of unfit elderly in clinical trials alongside the lack of a clear distinction between fit, frail, and vulnerable [32]. Major guidelines recommend less-intensive regimens or monotherapy in cases of comorbidities or expected poor tolerance [32,51].

Capecitabine monotherapy is effective and well tolerated in elderly mCRC patients unfit for combination regimens, even when very old (≥80 years) and multimorbid. Also, the oral route and the three-weekly schedule diminish the number of hospital accesses, thus facilitating the acceptance of treatment by the patient and reducing the burden on caregivers [35]. It is important to remember that capecitabine toxicity is increased with impaired renal function, which is common among the elderly. A reduced dose is required in case of mild renal insufficiency, while it is contraindicated when creatinine clearance is below 30 mL/min.

#### 4.1.2. Anti-VEGF

Bevacizumab, an anti-VEGF monoclonal antibody, is currently indicated in addition to first-line chemotherapy for *RAS/BRAF*-mutated or right-sided MSS/pMMR mCRC patients. Capecitabine or 5-FU plus bevacizumab is the preferred treatment of patients deemed unfit for intensive treatments, regardless of tumor molecular profile and sidedness [32].

The phase III MAX trial historically demonstrated the benefit of adding bevacizumab to capecitabine (with or without mitomycin) as first-line treatment of mCRC patients unsuitable for combination chemotherapy, without a significant increase in AEs [52]. The age of the 471 enrolled patients ranged from 32 to 86 years, with 40% ≥70 years old and 21% ≥75. When compared to younger patients, a higher proportion of the elderly (≥70 years) were in worse general condition (ECOG PS = 2 in 12% vs. 5%, *p* = 0.008) and had relevant comorbidities, particularly hypertension and prior cerebrovascular and ischemic events. However, the significant gain in PFS of capecitabine plus bevacizumab compared to capecitabine alone was confirmed also in the elderly (HR = 0.53, *p* = 0.01), with a trend towards a better OS as seen in the overall population (HR = 0.80, *p* = 0.41), despite the lower dose of capecitabine administered to them. The interaction analysis based on age subgroups demonstrated that the effect of bevacizumab on survival was consistent across age categories (> vs. ≤75 years), without major differences in bevacizumab-related toxicities [5]. Nonetheless, no formal CGA was assessed, limiting the ability to accurately stratify frailty.

These findings were corroborated by the randomized phase III AVEX trial (N = 280), which prospectively evaluated capecitabine with or without bevacizumab as first-line treatment for mCRC patients aged ≥ 70 years unfit for intensive chemotherapy. The median age was 76 (range 70–87), and around two-thirds of the patients were ≥75 years old, with 20% ≥80. Nearly all the patients (>90%) were on concomitant medications for comorbidities; however, they had a good ECOG PS (0–1 in 91%). The combination significantly improved PFS (9.1 vs. 5.1 months; HR 0.53, *p* < 0.0001) and showed a trend toward improved OS (20.7 vs. 16.8 months, *p* = 0.18). The benefit was consistent across age subgroups (70–74 years, 75–79, and ≥80) and no significant interaction for survival was found based on age (< vs. ≥75 years) or ECOG PS (0 vs. ≥1), although no formal CGA was performed [44].

A pooled analysis of four randomized trials confirmed the benefit of bevacizumab added to chemotherapy (5-FU or capecitabine, FOLFIRI, FOLFOX/XELOX) regardless of age. Out of 3007 included patients, 2293 were treated in the first-line setting, with 40% (N = 925) ≥65 years, and 25% (N = 582) ≥70 years. Bevacizumab in first line relevantly improved PFS across all age groups (<65 years: HR 0.60; ≥65 years: HR 0.59; ≥70 years: HR 0.54), while the magnitude of benefit in terms of OS diminished throughout age cohorts, thus remaining statistically significant (<65 years: HR 0.79, 95% CI 0.69–0.89; ≥65 years: HR 0.86, 95% CI 0.74–1.00; ≥70 years: HR 0.80, 95% CI 0.67–0.97). The higher rate of non-cancer-related deaths among the elderly may have hampered OS results [53].

Overall, fluoropyrimidine monotherapy with or without bevacizumab showed an acceptable safety profile, also when analyzed by age groups, with an expected increase in thromboembolic events and other anti-VEGF-related toxicities (such as arterial hypertension) with bevacizumab [5,44,53]. When compared to the youngers, patients aged ≥ 65 years appear more prone to arterial and venous thromboembolic events regardless of treatment and the risk of bevacizumab-related arterial thromboembolism seems to increase with the age (<65 years: 2% with or without bevacizumab; ≥65: 5.7% with and 2.5% without; ≥70: 6.7% with and 3.2% without) [53,54]. In the phase II PRODIGE 20 study, whose 102 elderly (≥75 years) mCRC patients receiving first-line chemotherapy plus or less bevacizumab underwent CGA at baseline and at each evaluation, the addition of bevacizumab did not negatively affect the maintenance of QoL and autonomy, and its known benefit was retained in patients with unfavorable baseline CGA scores. Independence in IADL was predictive of good efficacy and a favorable safety profile at multivariable analysis (*p* = 0.031) for both treatment arms, while the other geriatric variables had no impact on oncologic outcomes [55].

In contrast, the role of oxaliplatin added to fluoropyrimidine plus bevacizumab remains uncertain in older patients, with conflicting results likely depending on the level of frailty. Actually, prospective results from small, single-arm phase II trials evaluating oxaliplatin-doublets plus bevacizumab among elderly (≥70 or ≥75 years) mCRC patients showed that oncological outcomes were comparable to those expected from previous studies, including all-age mCRC patients (ORR around 50%, mPFS around 11 months, and mOS around 21 months), without unexpected safety signals [35,56,57,58]. In the AXELOX trial, whose 48 patients were half fit and half vulnerable, no differences in terms of safety, ORR, and PFS emerged between the two groups, while OS of the vulnerable was numerically shorter (17.4 vs. 20.7 months) [58]. Similarly, post hoc analysis from phase III randomized trials showed similar results in terms of efficacy, safety, and impact on QoL of oxaliplatin-doublet plus bevacizumab between younger and fit older patients (< vs. ≥70 years), despite the higher probability of dose reduction or treatment suspension reported among the elderly [54,59].

On the opposite, the phase III randomized JCOG1018 (RESPECT) (N = 251) trial failed to show an improvement in PFS (HR = 0.80, *p* = 0.09) and OS (HR 1.05, *p* = 0.69) with the addition of oxaliplatin to fluoropyrimidine and bevacizumab as first line treatment of unfit elderly (70–74 years with ECOG PS 2 and ≥75 years with ECOG PS 0–2) mCRC patients, despite the significantly higher ORR in oxaliplatin arm (47.7% vs. 29.5%, *p* = 0.006). The majority of patients were at least 75 years old (75–79: 45%; 80–84: 38%; ≥85: 13%), and in fair general condition (ECOG PS 2: <10%; VES-13 ≥3: <35%). Baseline CGA was missing in 20% of patients. Efficacy results were consistent across subgroups, including age (70–79 or ≥80 years) and geriatric score (VES-13 > or ≤3). Patients receiving oxaliplatin more frequently bore high-grade AEs and discontinued treatment because of toxicities [60].

The addition of irinotecan to first-line 5FU plus bevacizumab has been explored to a lesser extent among elderly mCRC patients. However, the benefit of irinotecan-based treatment among the elderly (≥65 and >70 years) appears similar to younger patients [61,62] though with an increased risk of neutropenia and asthenia [62,63].

According to previous promising results from the TASCO1 study [64], an alternative first-line regimen for unfit mCRC patients was proposed in the phase III SOLSTICE trial, which compared capecitabine with trifluridine-tipiracil (FTD/TPI), both in combination with bevacizumab. Out of 856 total patients, 60% were ≥70 years old, 45% ≥75, and a relevant proportion were in modest general condition (ECOG PS = 2: 18%; CCI ≥ 3: around 12%; G8 ≥ 14: around 50%). No differences in terms of PFS (HR 0.87, *p* not significant according to protocol design), OS (HR 1.06), and ORR (*p* = 0.09) were observed. However, an improvement in PFS was reported among patients with a CCI of 0 and ≥70 years, and those with G8 ≥ 14 treated in the FTD/TPI arm, suggesting that FTD/TPI plus bevacizumab might be an option for fit elderly patients. A higher incidence of G3–4 AEs was associated with FTD/TPI treatment (87% vs. 66%) without difference in QoL; FTD/TPI was more commonly associated with neutropenia and anemia, while capecitabine was linked to a higher incidence of hand-foot syndrome [65].

The superiority of an intensified chemotherapy backbone, FOLFOXIRI plus bevacizumab, in terms of PFS, OS, and ORR as a first-line treatment for mCRC patients was confirmed by an individual patient data meta-analysis of 5 phase II and III trials. The median age of the 1705 patients included in the meta-analysis was 61 (range 53–67), with 15% (N = 268) aged 70 years or more. The superiority in terms of activity and efficacy of FOLFOXIRI plus bevacizumab was preserved across age subgroups (< or ≥70 years) [66]. Accordingly, in a pooled analysis of TRIBE and TRIBE-2 trials accounting for 1187 mCRC patients with 15% (N = 182) fit elderly (70–75 years), the benefit of the intensified regimen was similar in young and old patients (< or ≥70 years) without interaction between age and treatment effect [63]. Due to the increase in AEs, particularly nausea, diarrhea, neutropenia, and febrile neutropenia, FOLFOXIRI plus bevacizumab is suitable for selected patients with right-sided and/or *RAS*-mutated, MSS/pMMR mCRC, including elderly patients in good general condition (ECOG PS = 0) and up to 75 years old [66]. Actually, elderly patients are at higher risk of high-grade toxicities (73% vs. 60%; *p* < 0.01), above all diarrhea and febrile neutropenia, and elderly females seem to be more prone to every-grade and severe AEs, with febrile neutropenia exceeding the threshold of 10% (OR: 1.90, *p* < 0.001) [63,67]. Therefore, special caution should be used when administering FOLFOXIRI plus bevacizumab to elderly patients: starting at a reduced dose of 5-FU and irinotecan and prescribing granulocyte colony-stimulating factor (G-CSF) as primary prophylaxis could increase tolerability while preserving treatment delivery and efficacy [63,67].

Based on those data, a fluoropyrimidine plus bevacizumab is still the preferred choice as first-line treatment of elderly MSS/pMMR mCRC patients, particularly unfit ones. Also, molecular profiles could help refine treatment selection as alternative first-line options are available for elderly *RAS* wild-type mCRC patients. However, a CGA remains essential to ensure treatment is tailored to the individual’s clinical and functional profile, allowing some selected fit elderly patients to receive more intensive treatment, similar to younger ones, such as a doublet or even a triplet chemotherapy plus bevacizumab.

#### 4.1.3. Anti-EGFR

Doublet chemotherapy plus an anti-EGFR (panitumumab or cetuximab) is the recommended first-line treatment of left-sided *RAS/BRAF* wt, MSS/pMMR mCRC patients [32].

Recent meta-analyses, including key head-to-head trials comparing an anti-EGFR to bevacizumab, both added to first-line chemotherapy, strengthened the predictive role of primary tumor location in driving the choice of monoclonal antibody class when treating *RAS* wt mCRC patients, finally confirming the indication of chemotherapy plus an anti-EGFR for left-sided patients, while bevacizumab is the recommended choice for right-sided ones, despite the treatment goal [68,69].

The PARADIGM trial (N = 604) recently confirmed the superiority in terms of OS of the anti-EGFR panitumumab compared to bevacizumab, both added to FOLFOX as first-line treatment for RAS wt mCRC patients, particularly the left-sided ones. A relevant proportion (56%) of patients were at least 65 years old. The OS benefit was consistent across clinical subgroups, including age (< or ≥65 years), which was a stratification criterion [70].

In addition, the efficacy of anti-EGFRs among the elderly was evaluated in a pooled analysis of seven randomized phase II and III trials, which accounted for a total of 1920 *RAS* wt mCRC patients receiving doublet chemotherapy with or without an anti-EGFR in the first-line setting. Compared to younger patients, patients aged over 69 years (23%) were likely to present with a mediocre performance status (ECOG PS = 1 in 53% vs. 43%, *p* = 0.004) and a right-sided primary tumor (34% vs. 24%, *p* = 0.005). When restricting the analysis to the left-sided mCRC patients receiving chemotherapy plus an anti-EGFR (N = 669), no differences in terms of OS (25.6 vs. 30.3 months, *p* = 0.09) or PFS (9.0 vs. 11.2, *p* = 0.52) were found between older and younger patients after adjusting for clinically significant covariates. Safety profile was similar between younger and older patients, without relevant differences in high-grade AEs [6].

Regarding the specific group of unfit and/or very old patients, the benefit of adding 5-FU with or without oxaliplatin to an anti-EGFR is less clear.

The phase II PANDA trial provided substantial evidence in this setting by randomizing 185 elderly patients (70–75 years with ECOG PS ≤ 2 or ≥75 years with ECOG PS ≤ 1) to receive panitumumab plus FOLFOX (arm A) or plus 5-FU/LV (arm B) in a non-comparative manner. 5-FU bolus was omitted in both arms. Overall, the median age was 77 years (range 70–86), the majority of patients were >75 years old (60%) and had an unfavorable baseline CGA (ECOG PS ≤ 2: around 50%; G8 ≤ 14: almost 70%; medium-high or high CRASH score: 46%). At the unplanned comparison, the addition of oxaliplatin increased the chance of an objective response (69% vs. 52%, *p* = 0.018) without a corresponding survival difference (HR for PFS 1.08, *p* = 0.611; HR for OS 1.00, *p* = 0.986). Patients aged 70–75 years derived better OS from arm A, while the elderly performed better without oxaliplatin. While patients with worse G8 or CRASH score had sensibly shorter OS compared to patients in better general condition (mOS: 32.8 vs. 18.7 months, respectively, for G8 score > vs. ≤14, *p* < 0.001; 28.3 vs. 17.2 months, respectively, for low vs. medium-high/high CRASH score, *p* = 0.033), no interaction for PFS, OS, and ORR was found between treatment arm and CGA items. Toxicity was consistent with the expected drug profiles. Age (*p* = 0.83), G8 score (*p* = 0.34) and CRASH score (*p* = 0.22) were not predictive of high-grade AEs rate [71].

When focusing specifically on frail elderly patients, prospective trials evaluating an anti-EGFR alone or plus a fluoropyrimidine failed to generate solid evidence due to the difficulty in enrolling a consistent sample of patients with a generally unfavorable prognosis and requiring an important familiar support [72]. In some cases, the stringent eligibility criteria based on CGA hindered patients’ accrual, leading to premature trial stop [73]. Despite those limitations, first-line single-agent anti-EGFR appears to be safe and effective in frail and vulnerable *RAS* wt, left-sided mCRC patients [72,74].

Taken together, this evidence suggests the efficacy of anti-EGFR therapy even among the elderly when properly selected based on tumor molecular profile and sidedness. Moreover, fit elderly patients could be treated with a doublet plus an anti-EGFR, expecting a similar benefit as for the youngest, particularly when an objective response is required. Although anti-EGFRs are generally considered well tolerated, they bring peculiar AEs—particularly skin rash, diarrhea, and hypomagnesemia—that increase the toxicity burden of induction chemotherapy and persist during maintenance [75]. Therefore, after 4–6 months of induction, a switch to maintenance is desirable, and an anti-EGFR alone, even if inferior to anti-EGFR plus 5-FU/LV, is still a valuable option for the elderly as it allows a greater relief from AEs [71,76]. However, tolerance of both maintenance strategies appears similar between younger and older (< vs. ≥70 years) patients without differences in terms of safety profile and impact on QoL [77].

On the contrary, vulnerable elderly patients can be treated with an anti-EGFR plus 5-FU/LV to spare toxicities without compromising efficacy, while unfit patients should receive an anti-EGFR as a single agent only after a careful balance between potential risks and expected benefits.

Probably in the near future, a negative hyper-selection of MSS/pMMR mCRC based on molecular profile other than *RAS* and *BRAF* genes could help further refine elderly patients’ treatment on top of primary tumor location, expanding the administration of anti-EGFR to susceptible right-sided mCRC patients [78,79].

#### 4.1.4. Immunotherapy

Pembrolizumab is the current first-line treatment for MSI-H/dMMR mCRC patients; thus, the immune combination of nivolumab and ipilimumab is emerging as a better alternative to the current standard [32].

MSI-H/dMMR tumors account for only 5% of mCRC patients overall, while they increase up to 35% from 80 years of age [80]. Despite this, the elderly (≥70 years) constitute only a minority of patients enrolled in major clinical trials that led to ICI approval in first-line [81,82,83]. Although the traditional concept of immunosenescence suggests a decline in immune function with aging, recent observations indicate that older adults can still derive significant benefit from immunotherapy despite age-related changes in immune competence [84].

At the 5-year update of the KEYNOTE-177 trial, pembrolizumab continues to demonstrate superior efficacy, activity, and a more favorable safety profile compared to first-line standard chemotherapy in MSI-H/dMMR mCRC patients, alongside a substantial improvement in QoL. The median age of the 307 patients was 63 years (range 24–93), with almost half of the patients ≥ 65 years and 29% >70 years. Overall survival benefit was consistent across the prespecified key subgroups, including age (≤ or >70 years, *p* = 0.36) [82].

Following the results of a dedicated cohort of the phase II Checkmate-142 trial showing a profound activity and prolonged efficacy of first-line nivolumab plus ipilimumab [85] the phase III randomized Checkmate 8HW study demonstrated the superiority in terms of PFS of the immune-combo when compared to standard first-line chemotherapy and to single-agent nivolumab across all lines of treatment, particularly among patients with centrally confirmed MSI-H [82,83]. As expected, nivolumab plus ipilimumab increased the rate of G3–4 AEs compared to nivolumab alone (22% vs. 14%), thus remaining less toxic than chemotherapy (48%). The superiority of the experimental arm over chemotherapy was confirmed across all subgroups, in particular among the 82 (27%) patients aged 70 years or more, with a similar safety profile compared to younger patients [86]. Similarly, the benefit over nivolumab was preserved among subgroups, including age (< or ≥65 years [N = 261, 45%]).

Few real-world experiences focused on MSI-H/dMMR elderly mCRC patients receiving ICI(s). In particular, the Immuno-MSI observational trial prospectively evaluated immunotherapy as first or subsequent line for metastatic MSI-H/dMMR gastrointestinal patients, with a cohort focusing on the elderly (≥75 years). Out of 225 included patients, only 24 were elderly, accounting for 19 CRC plus 4 gastro-esophageal cancer patients. Older and younger patients experienced a similar incidence of immune-related AEs (irAEs), mostly of low-grade, both with single and double ICI treatment. The management of irAEs did not differ according to age in terms of treatment discontinuation and/or corticosteroid administration. Elderly patients derived similar benefit in terms of ORR, PFS, and OS when compared to younger ones, and no association between age and efficacy was found. However, CGA was not performed, and patients were mostly in good general condition (ECOG PS ≤ 1) [87]. A multicenter Italian dataset retrospectively evaluated the benefit of first-line ICI(s) among 67 elderly (≥70 years) MSI-H/dMMR mCRC patients. Out of 20 (30%) evaluated patients, the majority had a poor baseline G8 score (N = 17, 85%). A consistent proportion of patients was over 74 years (75–79 years: 27%; ≥80: 39%). Immunotherapy was safe, with mostly low-grade AEs and no G4–5 events, and effective, even among patients 80 years or older [88].

Taken together, these findings suggest that advanced age alone should not preclude the use of ICI(s) in patients with MSI-H/dMMR mCRC. Dedicated trials, or at least studies requiring baseline CGA for the elderly, are awaited to generate robust data on the real benefit of immunotherapy according to age and frailty category. Patients deemed frail at CGA may experience a higher risk of hospitalization and scarce survival even when treated with immunotherapy [89]. Actually, while the stratification into fit, vulnerable, and frail has been widely assessed to guide chemotherapy decisions, its applicability in the context of immunotherapy remains underexplored, reaffirming the need for further research [4].

The ongoing FOxTROT 5 trial, focusing on older and frail patients receiving dostarlimab for localized CRC, will hopefully bring wider knowledge [90].

### 4.2. Treatment After First-Line

Elderly mCRC patients progressing after first-line therapy have reduced chances to receive further treatments compared to the younger ones, as there is a lack of direct evidence of benefit from continuing active treatments after first progression. However, a retrospective pooled analysis of major prospective trials showed that younger and older patients (≤ or >70 years) derive similar benefits from second lines in terms of time to progression and OS. Age was not predictive of worse outcomes, even when patients were divided by subsequent decades of age. However, the elderly enrolled in those trials were still a minority (22%), with few patients of 80 years or more [91].

Table 3 summarizes recommendations for second- and further-line treatments.

#### 4.2.1. Subsequent Lines for “All-Comers”

Continuous angiogenesis inhibition throughout treatment lines is an effective strategy for mCRC patients [32].

Observational trials initially suggested the advantages and safety of continuing the administration of bevacizumab beyond progression (BBP) to first line [92,93]. In particular, the BRiTE study (N = 1953) prospectively analyzed a real-life cohort of mCRC patients receiving bevacizumab in the first-line setting, with 27% aged 65–74 years and 19% ≥75 years. Overall, first-line and on-bevacizumab PFS were comparable among age cohorts (around 10 months), while younger patients experienced numerically longer mOS (26, 21.1, and 19.2 months, respectively, for <65, 65–74, and ≥75 years). Patients receiving BBP achieved better survival after first progression compared to chemotherapy alone, regardless of age subgroups (< vs. ≥65 years). An expected higher rate of arterial thromboembolic events among the elderly was reported (4.1% ≥ 75 years, 2.0% < 75) without any other difference in AEs according to age. It should be recalled that the risk of incidence and worsening of arterial hypertension increases with prolonged bevacizumab exposure [92,94].

The advantage of continuing bevacizumab beyond first-line progression compared to chemotherapy alone was confirmed in prospective trials, and the benefit was consistent across all subgroups, including age (< vs. ≥65 years) [95,96].

The VELOUR trial (N = 1226) demonstrated that VEGF-trap aflibercept, added to second-line FOLFIRI, is active and effective in patients with mCRC who have progressed to an oxaliplatin-based chemotherapy, including those previously treated with bevacizumab [97]. A survival benefit was also observed in the elderly subgroup (≥65 years), with an absolute gain of 2 months in mPFS (6.6 vs. 4.4 months, HR 0.75) and 1.3 months in mOS (12.6 vs. 11.3 months, HR 0.85), similar to the youngest subgroup. No interaction for survival was found between treatment and age. The addition of aflibercept increased chemotherapy toxicities overall and even more among the elderly (G3–4 AEs with aflibercept: 89 and 80%, respectively, < and ≥65 years), particularly with an augmented risk of dehydration (*p* = 0.006), while the incidence of anti-angiogenic related toxicities was not influenced by age. It should be noted that only 36% (N = 443) of patients were at least 65 years old, and a minority (16%) were 75 years old or more [98].

However, the prospective observational study QoLiTrap, that included a total of 1277 patients with a substantial portion of elderly (21% [N = 227] aged 65–69 years; 20% [N = 259] 70–74 years; and 18% [N = 233] ≥ 75 years), confirmed the activity and efficacy of aflibercept plus FOLFIRI regardless of age, alongside the maintenance of QoL [88]. A short induction phase followed by maintenance with 5FU/LV plus aflibercept is a suitable strategy among the elderly, both fit and unfit ones, as it allows a relief from severe toxicities without relevantly impairing oncological outcomes [99].

In the RAISE trial evaluating ramucirumab added to second-line FOLFIRI following progression to bevacizumab plus an oxaliplatin based treatment, the addition of the anti-VEGFR2 agent significantly prolonged survival in the overall population, with a positive trend among the elderly (≥65 years, HR for PFS 0.82, *p* = 0.051; HR for OS 0.85, *p* = 0.16). No interaction was found between the treatment effect and age. Anti-angiogenic-related AEs did not increase among the elderly (≥65 or ≥75 years). Again, the elderly were only a minority of patients (≥65 years, 40% [N = 427]; ≥75, less than 10% [N = 92]) [100].

Recently, options for pretreated mCRC patients were enriched by the combination of FTD/TPI plus bevacizumab after the publication of the phase III SUNLIGHT trial, which demonstrated that adding bevacizumab to FTD/TPI improves OS and PFS across all subgroups, including patients previously treated with bevacizumab [101]. The benefit was consistent across all age groups (<65 years [56%], 65–74 [32%], and ≥75 [12%]), including the delay of ECOG PS worsening. A slightly higher incidence of G3–4 AEs was reported among 65–74 years old patients (<65 years: 69%; 65–74: 80%; ≥75: 67%), without difference in toxicity-related treatment interruption [102]. Real-world data indicate a widespread use of FTD/TPI plus bevacizumab in pretreated elderly patients. Notably, among 2369 patients treated with FTD/TPI plus bevacizumab included in a Japanese database, the majority (63%) were ≥65 years old, with 21% between 70 and 74 years, 14% between 75 and 79, and almost 9% 80 years or more. The efficacy of the combination was confirmed across all subgroups [103]. FTD/TPI plus bevacizumab resulted in effective and safe also among real-world patients deemed unsuitable for more intensive treatment due to comorbidities and/or age, even if the elderly (≥75 years, N = 62 [67%]) tended toward a shorter OS compared to younger ones (mOS 12.5 vs. 23.2 months, *p* = 0.068) [104].

FTD/TPI alone and regorafenib are standard options for heavily pretreated mCRC patients [50]. Both demonstrated superiority compared to placebo/best supportive care, with only a 2-month gain in OS, and without objective responses [105,106,107,108].

In the phase III RECOURSE trial, the median age of the 800 patients randomized to FTD/TPI or placebo was 63 years (range 27–82), with 44% [N = 352] aged 65 years or more. The survival benefit was consistent across subgroups, including age (< or ≥65 years) [105]. The subsequent prospective, observational PRECONNECT study, together with other real-world experiences from expanded access programs, confirmed those results, also showing that FTD/TPI efficacy was not influenced by age (≤ or >70 years) [109,110]. However, when elderly patients were stratified by baseline CGA in the dedicated T-CORE1401 trial, a difference emerged: patients with a favorable G8 score (N = 8) compared to unfit ones (N = 22) had longer mOS (*p* = 0.04) and higher DCR (*p* = 0.01) [111]. FTD/TPI toxicity profile consists of frequent yet manageable hematologic toxicities, mostly non-febrile G3–4 neutropenia, and a few gastrointestinal AEs [105]. Elderly patients may encounter more high-grade AEs, both hematologic and non-hematologic, leading more frequently to dose reductions than younger patients. However, the incidence of febrile neutropenia, which is the prevalent concern, remains rare [109,111,112].

In the phase III CORRECT trial, 760 patients were randomized to regorafenib or placebo. The majority (62%, N = 475) of patients were young, with a median age of 61 years (range 54–67). Out of 505 patients from the regorafenib arm, 31% were 65–74 years old, and less than 8% (N = 38) ≥75 years old. The regorafenib survival advantage was preserved across all subgroups, including age (< or ≥65 years). The most frequent AEs were hand-and-foot syndrome, fatigue, and diarrhea, even of high-grade. While in the CORRECT trial, the incidence of AEs was similar between young and old patients (< vs. ≥65 years) [107,113] the prospective observational CONSIGN study revealed a slight increase in toxicities with age (G ≥ 3 AEs: <65 years: 55%; 65–74 years: 59%; ≥75 years: 64%) that did not affect treatment delivery [114]. Several small phase II studies focused on elderly patients (≥70 years), even frail and vulnerable ones, revealing that regorafenib is still effective and safe [115,116]. However, treatment interruption due to AEs might be more common in older (≥80 years) and unfit (ECOG PS ≥ 1, impaired ADL) patients. Interestingly, the presence of a caregiver appears to be predictive of a better OS [115]. A dose escalating strategy improves regorafenib tolerance and QoL without affecting efficacy [116]. Among elderly (≥75 years) patients, a modified schedule (2 weeks on/1 week off) with a starting dose tailored to baseline CGA and age group showed an amelioration in tolerability with similar oncological outcomes, underscoring the importance of functional patient selection [117].

Network meta-analyses and propensity score analyses showed comparable results of regorafenib and FTD/TPI overall, even if regorafenib seems to have more favorable survival among young patients, whereas FTD/TPI among the elderly (< and ≥65 years, respectively) [118,119,120].

Recently, fruquintinib became another option for refractory mCRC patients who failed all available treatments, including regorafenib and/or FTD/TPI [32]. In the FRESCO-2 global phase III trial, fruquintinib prolonged OS by 2 months compared to placebo, confirming previous results from Chinese patients [121,122]. The median age of patients was 64 years (range 56–70), with almost half of the patients (47%, N = 325) being ≥65 years old. Results were consistent across all subgroups, including those with age (< or ≥65 years). Sixty-three percent of patients receiving fruquintinib suffered from high-grade AEs, mostly hypertension and fatigue. To increase tolerability among the elderly, a dose escalating strategy has been proposed in a small phase II Chinese trial including 29 patients aged 65 years or more, mostly in mediocre general conditions (ECOG PS = 1 in 86%, G8 ≤14 in 69%, nutritional assessment at risk in 90%). Half of the patients tolerated fruquintinib up to 4 mg/day, while only four patients were able to receive the full 5 mg/day dosage. Efficacy was in line with what was expected from phase III trials. Baseline CGA did not affect results; however, the small number of patients limits the generalizability of those findings [123].

#### 4.2.2. Targeted Treatments

- Anti-EGFR rechallenge

After failure of an anti-EGFR-free treatment, rechallenge with anti-EGFR antibodies represents an active strategy in *RAS/BRAF* wild-type mCRC patients who previously progressed while on treatment with panitumumab or cetuximab.

A liquid biopsy demonstrating the absence of acquired resistance mutations on ctDNA is essential to pursue this strategy. Principal trials prospectively investigating rechallenge based on liquid biopsy, such as PARERE, CHRONOS, and CITRIC, included a variable proportion of elderly patients, with age ranging up to 86 years [124,125,126].

Unfortunately, no age-specific subgroup analyses were reported. However, given the general good tolerability of anti-EGFRs among pretreated, even comorbid elderly mCRC patients [74,127], anti-EGFR rechallenge can be considered a suitable option as a later line in elderly patients properly selected on the basis of liquid biopsy. Dedicated studies or at least post hoc age-specific analyses of major trials are awaited to confirm that assumption.

- Targeting *BRAF* V600E mutation

Only a small part (10%) of mCRC patients harbor the *BRAF* V600E mutation, but its prevalence increases among the elderly, more typically females [128]. In recent years, the targeted treatment encorafenib plus cetuximab (EC) was approved for *BRAF* V600E-mutated mCRC patients who progressed to previous line(s) [32] after the phase III BEACON trial demonstrated its superiority in terms of ORR, OS, and PFS compared to an irinotecan-based chemotherapy. Out of 665 patients enrolled in the BEACON, 220 received a doublet targeted treatment. The median age was 61 years (range 30–91), with approximately 20% aged 70 or more. Survival advantage was preserved across all subgroups and regardless of age (< or ≥65 years), with a manageable safety profile [129,130]. Nevertheless, a post hoc subgroup analysis showed that high-grade AEs were significantly more common among the elderly (≥70 years: 72%; <70 years: 53%; *p* = 0.017), particularly nausea, vomiting, abdominal pain, and fatigue/asthenia [131]. It should be noted that no formal geriatric assessment was performed in the BEACON trial, and that patients were mostly in quite good condition (ECOG PS 0–1 in every but two patients); therefore, structured data on frailty or comorbidities, which could affect tolerability and management of more vulnerable elderly patients, are lacking. However, the absence of interaction between age and treatment efficacy was confirmed in real-world studies, where patients with a higher median age and/or worse general condition are more represented [132,133].

The treatment algorithm for *BRAF* V600E-mutated mCRC patients is rapidly evolving.

The randomized phase III trial BREAKWATER recently demonstrated the outstanding superiority of EC plus FOLFOX compared to standard of care as first-line treatment of *BRAF* V600E-mutated mCRC, meeting the dual primary end point of ORR (66% vs. 37%, *p* = 0.0008) and OS (30.3 vs. 15.1 months, HR 0.49, *p* < 0.001), together with PFS (12.8 vs. 7.1 months, *p* < 0.001). Even if the median age was 61 years, patients up to 84 years old were enrolled. The advantage of EC plus FOLFOX was consistent across all prespecified subgroups, including age (< or ≥65 years). As expected, G3–4 AEs increased in the experimental arm (81.5% vs. 67%), making the combination less appealing for patients who are unfit or elderly. In this context, EC alone emerged as a suitable alternative to standard chemotherapy even in the first-line setting, given the numerically better oncological outcomes (OS 19.5 months, PFS 6.8 months, ORR 46%) with a more manageable safety profile (G3–4 AEs: 42.5%) compared to standard of care. Results of EC plus FOLFIRI are not mature yet [134,135]. Therefore, in the near future, previously untreated elderly patients with *BRAF* V600E-mutated mCRC will probably receive EC with or without doublet chemotherapy according to their level of frailness.

- Other targeted treatments

*KRAS G12C* mutations or *HER2* amplification represent a small subset (each <5%) of mCRC patients. ESMO guidelines have recently included anti-KRAS G12C [ESCAT I-A] and anti-HER2 [ESCAT II-B] directed therapies in the therapeutic algorithm of previously treated mCRC patients [32].

HER2 amplification is more commonly found in *RAS* wt and left-sided mCRC patients, and it is likely related to a poorer benefit from anti-EGFR-based treatments. Many anti-HER2 agents have been explored in HER-positive (IHC 3+ or 2+ with ISH amplified) refractory mCRC patients, with promising results (ORR ranging approximately from 20 to 40%; mPFS up to 8 months; and mOS around one year in most studies) [136]. More recently, in the trial Destiny-CRC02, the antibody-drug conjugate (ADC) trastuzumab-deruxtecan achieved similar results among HER2-positive mCRC patients, including patients previously treated with a HER2-targeted therapy, *RAS*-mutated, and regardless of primary tumor location [137]. In the cohort A of the previous phase II Destiny-CRC01 trial (N = 53), anti-tumor activity of trastuzumab–deruxtecan was preserved across age subgroups (<65 [N = 35] or ≥65 years [N = 18]; median age 58.5, range 27–79) [138]. However, the median age of patients enrolled in major anti-HER2 trials was lower than 60 years, and most of the studies did not show age-specific analyses, thus limiting the possibility of generalizing the results to the elderly population. Also, the known cardiotoxic class-effect of anti-HER2 agents, together with the emerging pulmonary toxicity of ADCs, might be a major concern among the elderly, as already seen for breast cancer patients [139].

*KRAS* G12C mutations are associated with unsatisfactory response to standard chemotherapy and worse OS than non-G12C *KRAS* mutations [140]. The CodeBreaK 300 trial, combining the anti-KRAS G12C sotorasib at 960 mg/day or 240 mg/day with the anti-EGFR panitumumab, has been the first phase III randomized trial to demonstrate the superiority of a KRAS inhibitor plus an anti-EGFR over standard treatment in refractory mCRC patients. The median age of the 160 randomized patients was 62 years (range 34–82), with 40% aged 65 years or more. In the subgroup analyses, the survival benefit was preserved among older patients (≥65 years) with both sotorasib dosages [141,142]. The combination of other anti-KRAS G12C, such as adagrasib, divarasib, or garsorasib, with the anti-EGFR cetuximab achieved similar results in non-randomized phase I or II trials [140]. Trials investigating KRAS G12C targeted therapies included patients up to 87 years old [143]; however, the median age was lower than 65 years, and age-specific subgroups analysis is generally not reported. The safety profile is similar among different KRAS G12C inhibitors, consisting mainly of low-grade gastrointestinal AEs and skin rash [130]. Tolerance appeared similar between younger and older (<65 vs ≥65 years; <75 vs ≥75 years) patients receiving sotorasib alone for non-small cell lung cancer [134].

Other, rarer mutations with available targeted therapies can be found in mCRC patients, and some of them seem to be more common among the elderly. In particular, *NTRK* fusions (0.2–2.4% of mCRC), *ROS1* and *ALK* rearrangements (<2% of mCRC) are enriched in right-sided, *RAS* wt, MSI-H/dMMR tumors, particularly in elderly patients. They confer poor prognosis and resistance to anti-EGFR-based treatments, but they can be effectively addressed with tumor-agnostic approved targeted inhibitors [144,145,146]. Given the rarity of those mutations, we will likely never have enough data on administering specific targeted agents to elderly mCRC patients. In addition, elderly cancer patients have lower chances of being offered an extended molecular profiling [147,148]. Leaving the genomic profile of elderly patients unknown could preclude them from a highly effective targeted treatment with an expected better tolerability than standard chemotherapy due to the absence of direct cytotoxic effects.

## 5. Conclusions

The management of older CRC patients is complex and requires a multidimensional approach. Age per se should not be the only factor leading treatment decisions. A CGA more accurately depicts the patient’s overall status, allowing for the prediction of overall survival due to pre-existing conditions and comorbidities, the estimation of resilience to treatment toxicities, and, ultimately, the guidance of an appropriate oncological intervention. Applying CGA at baseline and at disease re-evaluations could really help tailoring treatment in clinical practice: elderly fit patients should be treated similarly to their younger counterparts, even if a more cautious approach (e.g., starting chemotherapy at reduced dose; switching earlier to maintenance; or reinforcing ancillary medication) can be applied; vulnerable patients can be safely and efficaciously treated with less intensive therapy; frail patients are suitable for monotherapy or best supportive care alone.

Alongside precision medicine, oncologic care of the elderly must evolve toward a truly geriatric-informed approach. The implementation of CGA is highly advised for clinical practice. The time-consuming limitations of a comprehensive assessment can be overcome by a two-step approach: starting with a screening tool that can be administered by a trained nurse, and then scheduling an appointment with a designated geriatrician for patients at risk. In future trials dedicated to the elderly, the baseline CGA might become a mandatory inclusion or even a stratification criterion. In the meantime, studies that include patients over 70 years of age should require at least a baseline rapid geriatric screening (e.g., G8 score) to identify the elderly who are deserving of further geriatric evaluation. In addition, the systematic evaluation of treatment impact on QoL in clinical trials enrolling elderly patients should be implemented by using standardized questionnaires specifically developed for them in order to distinguish age-related decline from negative effects of therapy.

This integrated management aims to optimize efficacy, reduce treatment-related burden, and improve survival and quality of life.

## Figures and Tables

**Table 1:** Adjuvant Treatment based on CGA stratification.

Stage of Disease	Geriatric Status	Recommended Approach	Notes
High-risk stage II	Fit	Observation or FP monotherapy for 6 months in high-risk cases with pMMR.	No clear OS benefit with oxaliplatin.
	Vulnerable	- Observation. - Evaluate FP monotherapy for six months in high-risk cases with pMMR.	Oxaliplatin not recommend due to the increased risk of toxicity.
	Frail	Observation.	No indication for adjuvant chemotherapy; risk > benefit.
Stage III	Fit	XELOX for 3 months (low risk) or XELOX/FOLFOX for 6 months (high risk). In case of toxicities, consider oxaliplatin discontinuation after 3 months for pMMR or treatment interruption for dMMR.	Similar benefits to younger patients.
	Vulnerable	- pMMR: FP monotherapy for 6 months, particularly in high-risk cases. - dMMR: observation.	Oxaliplatin not recommended, especially if not well compensated.
	Frail	- Observation. - Evaluate FP monotherapy at reduced dose only for high-risk cases with pMMR.	Increased probability of discontinuation even with monotherapy.

Legend: CGA, comprehensive geriatric assessment; FP, fluoropyrimidines; OS, overall survival; p/d MMR: proficient/deficient mismatch repair.

**Table 2:** First-line treatment for metastatic disease based on CGA stratification.

Geriatric Status	Objectives	Recommended Approach	Notes
Fit	As for younger patients	As for younger patients. Consider starting at a reduced dose with subsequent increase if well tolerated. Consider G-CSF primary prophylaxis, particularly for women, bone metastases, and previous radiotherapy. - Left-sided, *RAS/BRAF* wt: doublet CT + anti-EGFR - Right-sided and/or *RAS/BRAF* mut: doublet CT + bevacizumab; FOLFOXIRI + bevacizumab	Similar effectiveness to young patients. FOLFOXIRI+ bevacizumab: only for 70–75 years old patients with ECOG PS = 0.
Vulnerable	PFS prolongation, independence preservation, QoL maintenance	Reduced dose is advisable. Consider the short duration of induction CT followed by maintenance - Left-sided, *RAS/BRAF* wt: doublets CT at reduced dose + anti-EGFR; FP + anti-EGFR; anti-EGFR monotherapy; FP +/− bevacizumab - Right-sided and/or *RAS/BRAF* mut: FP +/− bevacizumab; eventual addiction of oxaliplatin with personalized schedule.	Requires dose adjustment and close toxicity monitoring. Early activation of simultaneous care.
Unfit	Symptoms control, QoL maintenance, avoidance of severe toxicity	- Left-sided, *RAS/BRAF* wt: FP monotherapy at reduced-dose or anti-EGFR monotherapy. - Right-sided and *RAS/BRAF* mut: FP monotherapy at reduced-dose +/− bevacizumab. - Supportive care alone is a valid alternative.	Avoid combination chemotherapy. Monitor renal function for capecitabine. Consider definitive interruption of active treatment in case of toxicities. Early activation of simultaneous care.

Legend: CGA, comprehensive geriatric assessment; G-CSF, granulocyte colony-stimulating factor; CT, chemotherapy; PFS, progression-free survival; QoL, quality of life; FP, fluoropyrimidines; WT, wild type; Mut, mutated.

**Table 3:** Second and further lines of treatment based on CGA.

Treatments After First-Line			
Geriatric Status	Objectives	Recommended Approach	Notes
Fit	As for younger patients	Administer second and further lines of treatment as indicated for younger patients, based on previous treatment and according to disease characteristics. Consider starting at a reduced dose with subsequent increase if well tolerated. Consider G-CSF primary prophylaxis, particularly for women with bone metastases and previous radiotherapy.	Reassess fitness with CGA before administering the second and further lines of treatment. Pay attention to peculiar AEs of targeted treatments. Activate simultaneous care.
Vulnerable	Minimize toxicity, contain symptoms, preserve QoL	Based on previous treatment and according to disease characteristics. Prefer chemo-free regimens. In case of CT: start at reduced dose and/or prefer monotherapy; consider short induction CT and early switch to maintenance. Dose escalation is also advisable for regorafenib, fruquintinib, and FTD/TPI +/− bevacizumab.	Pay attention to peculiar AEs of targeted treatments and to the appearance of cognitive decline. Activate simultaneous care. Consider definitive interruption of active treatments in case of relevant toxicities.
Frail	Maintain QoL, contain symptoms, avoid toxicity	Based on previous treatment tolerance. - Supportive care alone is a valid option. - Monotherapy (preferably chemo-free regimens and oral route) according to disease characteristics and previous treatments.	The presence of a caregiver and the early activation of simultaneous care are essential. Consider active treatments when the amelioration of cancer symptoms is required, with definitive interruption in case of toxicities.

Legend: CGA, comprehensive geriatric assessment; G-CSF, granulocyte colony-stimulating factor; CGA, comprehensive geriatric assessment; QoL, quality of life; FTD/TPI Trifluridin/Tipiracil; CT, chemotherapy; AEs, adverse events.

## Supplementary Materials

The following supporting information can be downloaded at: https://www.mdpi.com/article/10.3390/cancers17203336/s1, Checklist S1: JBI’s critical appraisal checklist for textual evidence.
