# Supplementary material for: Management of Elderly Colorectal Cancer Patients: A Comprehensive Review Encompassing Geriatric Assessment
Source: Cancers (Basel). 2025 Oct 16;17(20):3336. doi: 10.3390/cancers17203336 (PMC12564925; doi:10.3390/cancers17203336)
Supplement: Supplementary file 1 [file cancers-17-03336-s001.zip › cancers-3885379-supplementary.pdf]

CHECKLIST FOR TEXTUAL EVIDENCE: NARRATIVE

Critical Appraisal tools for use in JBI Systematic Reviews

CRICOS Provider Number 00123M

INTRODUCTION

JBI is an international research organisation based in the Faculty of Health and Medical Sciences at the University of Adelaide, South Australia. JBI develops and delivers unique evidence-based information, software, education and training designed to improve healthcare practice and health outcomes. With over 70 Collaborating Entities, servicing over 90 countries, JBI is a recognised global leader in evidence-based healthcare.

JBI Systematic Reviews

The core of evidence synthesis is the systematic review of literature of a particular intervention, condition or issue. The systematic review is essentially an analysis of the available literature (that is, evidence) and a judgment of the effectiveness or otherwise of a practice, involving a series of complex steps. JBI takes a particular view on what counts as evidence and the methods utilised to synthesise those different types of evidence. In line with this broader view of evidence, JBI has developed theories, methodologies and rigorous processes for the critical appraisal and synthesis of these diverse forms of evidence in order to aid in clinical decision-making in healthcare. There now exists JBI guidance for conducting reviews of effectiveness research, qualitative research, prevalence/incidence, etiology/risk, economic evaluations, textual evidence, diagnostic test accuracy, mixed-methods, umbrella reviews and scoping reviews. Further information regarding JBI systematic reviews can be found in the JBI Evidence Synthesis Manual.

JBI Critical Appraisal Tools

All systematic reviews incorporate a process of critique or appraisal of the research evidence. The purpose of this appraisal is to assess the methodological quality of a study and to determine the extent to which a study has addressed the possibility of bias in its design, conduct and analysis. All papers selected for inclusion in the systematic review (that is – those that meet the inclusion criteria described in the protocol) need to be subjected to rigorous appraisal by two critical appraisers. The results of this appraisal can then be used to inform synthesis and interpretation of the results of the study. JBI Critical appraisal tools have been developed by the JBI and collaborators and approved by the JBI Scientific Committee following extensive peer review. Although designed for use in systematic reviews, JBI critical appraisal tools can also be used when creating Critically Appraised Topics (CAT), in journal clubs and as an educational tool.

© JBI, 2020. All rights reserved. JBI grants use of these tools for research purposes only. All other enquiries should be sent to jbisynthesis@adelaide.edu.au.

JBI CRITICAL APPRAISAL CHECKLIST FOR TEXTUAL EVIDENCE: NARRATIVE

Reviewer_______________________________________Date_______________________________

Author____Year_________ Record Number_________

	Yes	No	Unclear	Not applicable
1. Is the generator of the narrative a credible or appropriate source?	□	□	□	□
2. Is the relationship between the text and its context explained? (where, when, who with, how)	□	□	□	□
3. Does the narrative present the events using a logical sequence so the reader or listener can understand how it unfolds?	□	□	□	□
4. Do you, as reader or listener of the narrative, arrive at similar conclusions to those drawn by the narrator?	□	□	□	□
5. Do the conclusions flow from the narrative account?	□	□	□	□
6. Do you consider this account to be a narrative?	□	□	□	□

Overall appraisal: Include □ Exclude □ Seek further info □

Comments (Including reason for exclusion)

____

____

____

© JBI, 2020. All rights reserved. JBI grants use of these tools for research purposes only. All other enquiries should be sent to jbisynthesis@adelaide.edu.au.

EXPLANATION OF TEXTUAL EVIDENCE: NARRATIVE CRITICAL APPRAISAL TOOL

How to cite: *McArthur, A.; Klugarova, J.; Yan, H.; Florescu, S. Chapter 4: Systematic reviews of text and opinion. In: Aromataris E, Munn Z (Editors). JBI Manual for Evidence Synthesis. JBI, 2020.*

Answers: Yes, No, Unclear or Not/Applicable

1. Is the generator of the narrative a credible or appropriate source?

It is important to establish the legitimacy of the narrator as part of assessing the degree to which the narrative is authentic. ***Ask:***

- Is this a first- hand account of an event?
- Do you sense that the author is both a credible and appropriate narrator?

2. Is the relationship between the text and its context explained?

Narrative always describes an event that occurs within a specific time and space; within a context. The relationship between the characters and the place in which the event occurs needs to be described. ***Ask:***

- Where does the event take place?
- Who does it involve?
- What occurs?

3. Does the narrative present the events using a logical sequence so the reader or listener can understand how it unfolds?

A narrative seeks to convince a reader; this, in assessing this narrative, the reviewer should 'follow' the narrative and its meanings. **Ask:**

- Can I 'imagine' the event, the characters involved and what happened?
- Does the 'story' or the account flow in a logical way?

4. Do you, as reader or listener of the narrative, arrive at similar conclusions to those drawn by the narrator?

Again, note the purpose of narrative to persuade or convince. **Ask:**

- Are the conclusions drawn from the description of the event?
- Are any seemingly causal relationships explained?
- Do you draw similar conclusion from the narrative as the narrator?

5. Do the conclusions flow from the narrative account?

Again, note the purpose of narrative to persuade or convince. **Ask:**

- Are the conclusions drawn from the description of the event?

© JBI, 2020. All rights reserved. JBI grants use of these tools for research purposes only. All other enquiries should be sent to jbisynthesis@adelaide.edu.au.

6. Do you consider this account to be a narrative?

In appraising the authenticity of the narrative, can you differentiate between the emotional persuasiveness of the 'story' with the objective accuracy of the narrative? **Ask:**

- What is the degree of narrativity in this piece?

© JBI, 2020. All rights reserved. JBI grants use of these tools for research purposes only. All other enquiries should be sent to jbisynthesis@adelaide.edu.au.
